# DELIVERING AND EVALUATING A VIRTUAL DYADIC BEHAVIORAL SLEEP INTERVENTION

**DOI:** 10.1093/geroni/igad104.1319

**Published:** 2023-12-21

**Authors:** Glenna Brewster, Jordan Pelkmans, Kenneth Hepburn

**Affiliations:** Emory University, Atlanta, Georgia, United States; Emory University, Nell Hodgson Woodruff School of Nursing, Atlanta, Georgia, United States; Emory University, Atlanta, Georgia, United States

## Abstract

Disturbed sleep is prevalent among persons living with cognitive impairment and dementia and their care partners. While there are few interventions for care partners to manage their sleep problems there is a paucity of interventions where both the person living with cognitive impairment and their care partner work together to improve their sleep outcomes. The aim of this presentation is to discuss how we recruited participants and delivered and evaluated caregiver and person living with cognitive impairment outcomes of a 4-week videoconferencing delivered cognitive behavioral therapy for insomnia intervention. Recruitment is time intensive but feasible. In general, both members of the dyad reported a reduction in their insomnia scores. Care partners supported the person living with cognitive impairment with completing the diaries. Qualitatively, care partners didn’t report much difficulty with completing the diaries. Dyads reported enjoying the relaxation and the pleasant activities. Among those who had pets, it was difficult to follow the instructions regarding not having them in bed. They also had difficulty with the cognitive therapy logs. Most persons living with cognitive impairment did not use the cognitive therapy logs. This pilot study of a 4-session videoconferencing-delivered cognitive behavioral therapy for insomnia intervention resulted in positive outcomes for persons living with cognitive impairment and/or dementia and their care partners and qualitative feedback provided ways to tailor the intervention for a larger study.

